# Modelling the pre-assessment learning effects of assessment: evidence in the validity chain

**DOI:** 10.1111/j.1365-2923.2012.04334.x

**Published:** 2012-11

**Authors:** Francois J Cilliers, Lambert W T Schuwirth, Cees P M van der Vleuten

**Affiliations:** 1 Centre for Teaching and Learning, Stellenbosch UniversityStellenbosch, South Africa; 2 Flinders Innovations in Clinical Education, Flinders UniversityAdelaide, South Australia, Australia; 3 Department of Educational Development and Research, University of MaastrichtMaastricht, the Netherlands

## Abstract

**OBJECTIVES:**

We previously developed a model of the pre-assessment learning effects of consequential assessment and started to validate it. The model comprises assessment factors, mechanism factors and learning effects. The purpose of this study was to continue the validation process. For stringency, we focused on a subset of assessment factor–learning effect associations that featured least commonly in a baseline qualitative study. Our aims were to determine whether these uncommon associations were operational in a broader but similar population to that in which the model was initially derived.

**METHODS:**

A cross-sectional survey of 361 senior medical students at one medical school was undertaken using a purpose-made questionnaire based on a grounded theory and comprising pairs of written situational tests. In each pair, the manifestation of an assessment factor was varied. The frequencies at which learning effects were selected were compared for each item pair, using an adjusted alpha to assign significance. The frequencies at which mechanism factors were selected were calculated.

**RESULTS:**

There were significant differences in the learning effect selected between the two scenarios of an item pair for 13 of this subset of 21 uncommon associations, even when a p-value of < 0.00625 was considered to indicate significance. Three mechanism factors were operational in most scenarios: agency; response efficacy, and response value.

**CONCLUSIONS:**

For a subset of uncommon associations in the model, the role of most assessment factor–learning effect associations and the mechanism factors involved were supported in a broader but similar population to that in which the model was derived. Although model validation is an ongoing process, these results move the model one step closer to the stage of usefully informing interventions. Results illustrate how factors not typically included in studies of the learning effects of assessment could confound the results of interventions aimed at using assessment to influence learning.

Discuss ideas arising from this article at ‘http://www.mededuc.com discuss’

## Introduction

Calls have been made for a greater role for theory in medical education research.^1^–^5^ A theory can be used to generate and frame research questions, which, in turn, should help to interrogate and further refine the theory. Theory can inform the interpretation of observations. Using theory also allows the findings of research to be linked to the work of others at a conceptual level. A variety of small advances can be consolidated with existing work, even from other fields, when the work is theoretically grounded, whereas work that is not grounded in theory ‘runs the risk of being superficial and non-cumulative’.^4^ Theory also allows the informed development and investigation of solutions to problems.

Cautionary notes have been sounded about the uncritical use of theory, however.^4^,^6^,^7^ Colliver^6^ cautions against using theory that is ‘little more than metaphor, not rigorous, tested, confirmed scientific theory’. Bordage^4^ exhorts that prior to adopting a conceptual framework, the evidence supporting that framework should be carefully examined. This raises questions about how a theory should be evaluated before it is utilised.

Prochaska *et al.*^8^ reviewed the writings of philosophers of science to compile a hierarchical set of 12 criteria by which to evaluate theory, models and frameworks. These criteria delineate an approach to evaluating theory; however, they also suggest an agenda for validating new theory. The hierarchical nature of the criteria supports the notion that validation is a process, not an event, and validity is a journey, not a destination.

**Figure 1:**
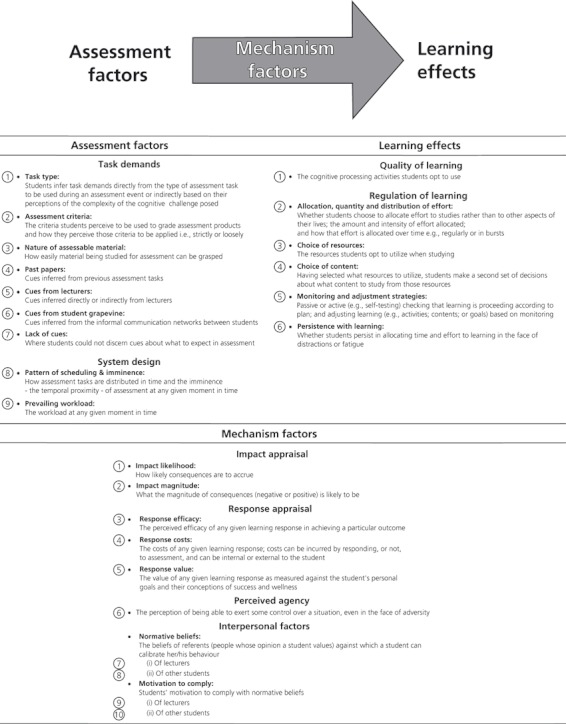
A model of the pre-assessment learning effects of consequential assessment for theory contexts. Nine assessment factors and six learning effects are linked in 40 associations^9^ (i.e. not all assessment factors and learning effects have been shown to be involved in associations with one another). Each assessment factor–learning effect pair is potentially linked in up to 10 different ways, depending on which one or more of a set of mechanism factors mediate any particular association. Which assessment factor(s) and mechanism factor(s) are at play – and therefore which learning effect(s) result – is influenced by personal and contextual factors

This paper is about one leg of a validity journey. Using grounded theory, we have proposed a model of the pre-assessment learning effects of consequential assessment (Fig. 1).^9^ Before addressing the central issue of model validity, some clarification of terminology is required.

‘Learning effects of assessment’: these effects have only recently been classified.^10^ Pre-assessment learning effects typically accrue to learning behaviours and occur before an assessment event (e.g. well-constructed multiple-choice questions drive learning for understanding before a test). Post-assessment learning effects occur after an assessment event and accrue to learning behaviours or subsequent achievement (e.g. feedback influences learning after a test and in-course assignments influence examination performance). Pure learning effects occur during an assessment event (e.g. learning is influenced by the testing effect^11^ or by constructing a portfolio).

‘Consequential assessment’: under consequential test conditions, stakes are typically high and assessment results in direct consequences for students, whereas under non-consequential test conditions, stakes are low and few or no consequences accrue.^12^ Although this may sound like summative and formative assessment, we have found that the stakes that influence learning in assessment situations encompass not only judgement-related academic consequences (typical of summative assessment), but also non-academic consequences (e.g. accruing to self-esteem or a sense of agency).^13^ This has led us to consider consequential assessment a better descriptor for the model than summative assessment.

Returning now to the issue of ‘validity’: before any model can meaningfully inform practice and research, its validity needs to be established. Interventions informed by substantiated theory are expected to be more successful than those that are not.^8^,^14^ There is currently no validated model explaining the pre-assessment learning effects of assessment. There is much literature describing what the pre-assessment learning effects of assessment are. However, a dominant theme in this literature has concerned whether a particular assessment task or programme induces a surface or deep approach to learning (e.g.^15^). Much less literature addresses the mechanism of this impact; most of this is fragmented (reviewed in 16). There have been few attempts to propose theoretical models to explain how pre-assessment learning effects come about.^12^,^17^–^21^ However, none of these models has been validated. Furthermore, none has gained traction to inform the practice of, or research about, assessment to induce sought-after pre-assessment learning effects, judging by citation patterns. This intriguing gap in the literature prompted us to develop and start to validate our model (Fig. 1).

As a first step in the validation process, we established that we could analyse and explain observations about the learning effects of assessment in a context different to that in which the model was derived.^13^ This was carried out qualitatively and with the respondent group that had been involved in the derivation of the model. The question now was how best to continue the model validation journey.

Given that the model had been derived qualitatively working with a small group of medical students in Years 4 and 5 at Stellenbosch University, we considered it a priority to explore whether the components and associations in the model were at play in a broader population of students. To forge the next link in the chain of validity evidence, we therefore elected to explore whether the model was operational more broadly among Year 4 and 5 medical students at Stellenbosch University.

A challenge in this endeavour concerned the large number of associations that could be explored (Fig. 1). The baseline qualitative work yielded 40 assessment factor–learning effect associations.^9^ The factors in each association are linked by up to 10 mechanism factors. This yields a total of 400 possible associations. However we chose to investigate these associations, it was not feasible to investigate all simultaneously. Doing so would have required impracticably long testing times of respondents.

The selection of a subset of associations to study was informed by literature on theory-building citing Popper’s maxim that theory must be falsifiable.^7^,^8^ Of the assessment factor–learning effect associations identified in the baseline work, 23 were classified as uncommon (i.e. there were fewer than five references to each in the entire dataset). These associations were considered least likely to be at play in a broader population. However, the corollary of this belief was that if these associations were at play in a broader population, this would provide stronger (if as yet partial) evidence in support of the model than would the demonstration that the 17 more common associations were at play. Therefore, as the most stringent test of the model, we opted to first focus on this subset of uncommon associations and essentially to try to refute their role in a broader population of students.

A subsequent challenge lay in conceptualising how best to explore the operation of the model. This process was informed by the fact that for any given student in any given assessment situation, learning effects result from the interplay between one or more assessment factors and one or more mechanism factors (Fig. 1). If the factors at play vary from one assessment scenario to the next, so too can the resultant learning effect(s). For example, a student may learn for understanding when assessment is a month away and appraisal of response value is the dominant mechanism factor at play, such that learning is geared towards helping patients. That same student may resort to memorisation when assessment is 3 days away, when appraisal of impact severity is the dominant mechanism factor at play, such that learning is geared towards avoiding failure in the course. Therefore, our research question was: in how many cases does varying the manifestation of an assessment factor result in a change in the ensuing learning effect and the main mechanism factor involved, for a subset of uncommon associations between assessment factors and learning effects? To investigate this, we undertook a cross-sectional survey using a purpose-made instrument in a population broader than but similar to that in which the model was derived.

## Methods

### Instrument

Developing a data collection instrument suitable for use with large numbers of respondents posed several challenges. We were working with an elaborate model developed from extensive descriptions of respondents’ lived experiences. We wanted to determine what a respondent’s learning response was in any given assessment scenario, what mechanism factor best explained that learning response in that scenario, and whether the learning response and the mechanism factor changed if the manifestation of the assessment factor was varied. This required an instrument that could both approximate assessment scenarios that respondents might find themselves in and explore respondents’ decision making for 23 different scenarios.

To address these challenges, we opted for low-fidelity simulation using a self-report instrument. We developed items based on situational judgement tests^22^ and incorporating the two-stage logic of the key features approach.^23^ We utilised data from the baseline qualitative study^9^,^16^ to construct pairs of closed items each comprising a vignette and two option sets (Table 1).

**Table 1:** Example of an item pair. This item pair addresses the influence of ‘cues from the student grapevine’ (assessment factor) on ‘quantity of effort’ (learning effect). Each vignette and the associated option sets were presented individually to respondents, not paired as illustrated

	Vignette 1	Vignette 2
Vignette depicting assessment factor	It is the middle of the first semester. You are at the start of a 4-week theory module. You have heard from students who did the module last year that the workload in the module is very high and that if you do not start studying early, you will never be adequately prepared for the test	It is the middle of the first semester. You are at the start of a 4-week theory module. You have heard from students who did the module last year that the workload in the module is lower than is usually the case and that you can safely spend time on things other than your studies during the module and still be adequately prepared for the test
Learning effect option set	When you decide how to spend your time this week, what are you most likely to do?	
	*Select the one choice that best applies*	
	(a) I will spend as much time as I usually do studying at this stage of a module	
	(b) I will spend more time than I usually do studying at this stage of a module	
	(c) I will spend less time than I usually do studying at this stage of a module	
Mechanism factor option set	Which of the following best explains your decision making?	
	*Although more than one option may be applicable, please select the one option that best applies to you*	
	(a) Nothing bad will happen to me academically if I do this	
	(b) There is too much at stake to risk doing badly or failing	
	(c) Doing this is necessary to get a result I’m comfortable with	
	(d) It’s not worthwhile letting my life get out of balance	
	(e) It is important to me to do well: I don’t want to do badly or fail	
	(f) I believe that I’m capable of doing well enough under these circumstances if I do this	
	(g) I have heard from other students that if I do this, I will do well enough	
	(h) Lecturers emphasise the importance of this	
	(i) It is worth taking guidance from my fellow students	
	(j) I value the opinion of lecturers who teach this module	

Each vignette described an assessment scenario revolving around one assessment factor. The manifestation of that assessment factor was varied from one item to the next in each pair. Based on the baseline qualitative data, the two manifestations of each assessment factor were selected in such a way that they were expected to produce different learning effects for the two scenarios.

The first option set depicted possible learning effects in the context of the vignette; the second depicted possible reasons for the behaviour (i.e. mechanism factors) in that context. Respondents were required to select the one option they felt best applied to them in that scenario from each option set. This allowed us to explore whether the learning effect and mechanism factor selected by a respondent in sets of paired scenarios changed as the manifestation of an assessment factor varied.

Of the 23 assessment factor–learning effect associations classified as uncommon, one was not studied. The curriculum had since been altered; contemporary respondents would not be able to relate to the scenario. Another item was discarded when it proved impossible to construct a plausible alternative scenario.

In some cases, it was not possible to create an alternative scenario by varying the assessment factor of interest, such as in the scenario that exhibited a ‘lack of cues’. Varying this would result in cues being given. It proved impossible to create a scenario in which the mere presence, rather than the nature, of those cues would be the influencing factor. In these cases, another factor was added (e.g. respondents were required to contemplate a scenario of no cues distanced in time from assessment and then with assessment imminent as the alternative scenario).

A total of 21 associations involving eight assessment factors and eight learning effects were thus investigated. The full item set is available from author FJC. Essentially, each item pair represented an individual study. These were grouped together in questionnaires for convenience.

In order not to contaminate the respondent pool and given that Year 3 students had insufficient experience of assessment methods for the purposes of the study, items were pilot-tested with six Year 6 medical students. The session lasted approximately 2 hours. Although changes were suggested and made to item wording, students found all scenarios to be realistic and all the options offered to be plausible. Students were given a voucher worth approximately US$20, but were not told ahead of time about this.

Based on how quickly students processed items in the pilot and to limit testing time to approximately 20 minutes, items were divided into two questionnaires of 10 and 11 pairs, respectively. The reading loads of the two questionnaires were comparable. Learning effect option sets were scenario-dependent. Items with similar learning effect options were placed consecutively in the questionnaire to limit the cognitive burden on respondents. To the same end, respondents were informed that mechanism factor option sets were identical for all scenarios.

Respondents self-reported their year of study, their average marks across the course up to that point, whether they had previously failed a module, and their gender.

### Population, data collection and ethical considerations

The study population comprised all medical students in Years 4 (*n* = 179) and 5 (*n* = 182) at Stellenbosch University (i.e. a population similar to but broader than that in which the model had been derived). Students followed a 6-year curriculum. Most students had entered medical school directly from secondary school.

Students were randomly allocated to complete Questionnaire 1 (*n* = 181) or Questionnaire 2 (*n* = 180). Items were administered electronically and anonymously using an in-house survey tool based on Checkbox Version 4.6.4.7 (Checkbox Survey Solutions, Inc., Watertown, MA, USA). All students in both classes were surveyed. The letter of invitation and the survey link were delivered using e-mail address lists used by faculty staff to communicate with students.

We implemented various recommendations to optimise response rates.^24^–^26^ These included offering students the incentive of entry into a draw for one of 20 vouchers to a vendor of their choice valued at approximately US$20 each if they participated in the study. Contact information for the draw was collected in a separate survey linked to the last page of the survey completed by the respondents. Informed consent was obtained from each participant prior to participation.

### Data analysis

Each item pair was, in essence, a mini-study: any item pair with responses to both scenarios could be included for analysis. There were more than two learning effect options for most items and therefore, for analysis, the response considered most likely to vary in the context of the scenario was identified using the baseline qualitative data. Selections of other response options were grouped to generate a 2 × 2 table. The chi-squared statistic was used to compare the frequency at which the learning effect considered most likely to vary was selected in the two variants of each scenario. The null hypothesis was that there would be no difference between the two scenarios in the frequency with which the learning effect most likely to vary was selected.

Given the large number of associations being explored, we applied Holm’s sequentially selective Bonferroni adjustment. The highest p-value considered to indicate significance was 0.00625. We also performed a sign test to determine whether the number of associations in which a significant difference in learning effect between scenarios was noted was greater than would be the case by chance alone.

We were also interested in the role of mechanism factors. The frequency at which any given mechanism factor was selected was calculated. We also calculated whether the mechanism factor selected changed from one scenario to the next for each item pair.

To determine how representative responders were of the study population, the chi-squared statistic was used to compare the two groups in terms of year of study, previous failure of a module, and gender. For each characteristic compared, the null hypothesis was that there would be no difference between the two groups. A p-value of < 0.05 was considered to indicate statistical significance.

Analyses were performed using statistica Version 10 (StatSoft, Inc., Tulsa, OK, USA)).

## Results

Questionnaires were completed by 152 respondents, yielding 80 complete responses to Questionnaire 1 and 72 to Questionnaire 2. In addition, 15 respondents provided responses to one or more item pairs, but did not complete a questionnaire. This resulted in varying numbers (between 74 and 84) of responses for each item pair and response rates of between 41.1% and 46.4%.

### Representativeness of respondents

Compared with the study population, greater proportions of Year 4 than Year 5 respondents than would have been expected responded to both Questionnaire 1 (*χ*^2^ = 7.52, d.f. = 1; p = 0.006) and Questionnaire 2 (*χ*^2^ = 7.29, d.f. = 1; p = 0.006). There were no differences between respondents and the study population in terms of gender and previous failure of a module. No comparisons were made for average scores as respondents tended to report their average scores in multiples of five. Scrutiny of the data in Table 2 suggests there is no difference.

**Table 2:** Demographic information for the study population and respondents

			Study population		Respondents	
				
Questionnaire	Programme	Failed ≥ 1 modules	*n*	Average score* (SD)	*n*	Average score^†^ (SD)
1	MBChB Year 4	No	68	69.3 (6.3)	42	69.5 (6.1)
		Yes	21	56.3 (4.0)	10	63.1 (6.7)
		Subtotal	89		52	
	MBChB Year 5	No	63	68.0 (5.3)	23	66.4 (4.7)
		Yes	29	57.3 (4.3)	12	61.5 (4.6)
		Subtotal	92		35	
1 Total			181		87	
2	MBChB Year 4	No	72	68.4 (6.8)	34	69.4 (7.7)
		Yes	18	55.9 (4.2)	15	59.7 (9.2)
		Subtotal	90		49	
	MBChB Year 5	No	59	69.6 (6.2)	20	68.9 (4.7)
		Yes	31	58.1 (4.6)	11	61.7 (4.0)
		Subtotal	90		31	
2 Total			180		80	
Grand total			361		167	

Average = average score across the entire programme

* Calculated from faculty records

† Self-reported

SD = standard deviation

### Association between assessment factors and learning effects

The results of what are essentially 21 separate studies are presented together for convenience. There were significant differences in the frequency at which the learning effect of interest was selected between the two variants of a scenario for 13 of the 21 scenarios (Table 3). This is greater than would be expected by chance (sign test, *Z* = 3.328201; p = 0.000874). The results therefore did not support the hypothesis that there would be no difference in the frequency at which the learning effect most likely to vary was selected between the two scenarios for most of this subset of uncommon associations between these factors.

**Table 3:** Change in learning effect and mechanism factor selected for each of the subset of uncommon assessment factor–learning effect associations investigated in this study. Each item pair investigated the association between one assessment factor, one learning effect and all 10 mechanism factors. For each item pair, the manifestation of the assessment factor varied from one scenario to the other. Each association investigated is depicted in the relevant cell in the table. Three sets of data are provided for each association: (i) the number of respondents who provided a complete response; (ii) LrnΔ: the χ^2^ statistic (d.f. = 1 for all) and associated p-value were calculated based on the frequency at which respondents selected the learning effect considered most likely to vary for the two variants of each scenario (*p-value significant at p < 0.00625), and (iii) MchΔ: the number (and percentage) of respondents who changed their choice of mechanism factor from one scenario to the other within the item pair

	Learning effects							
	
		Metacognitive regulation activities						
		
Assessment factors	Nature of cognitive processing activities	Allocation of effort: choice to learn	Quantity of effort	Distribution of effort	Choice of resources	Choice of content	Monitoring and adjustment strategies	Persistence with learning
Task demands								
Assessment criteria	*n* = 75 LrnΔ*χ*^2^ = 11.79 p = 0.0006* MchΔ 34/75 (45.3%)		*n* = 82 LrnΔ*χ*^2^ = 0.75 p = 0.3879 MchΔ 28/82 (34.1%)		*n* = 83 LrnΔ*χ*^2^ = 2.54 p = 0.1108 MchΔ 50/83 (60.2%)			
Nature of assessable material		*n* = 74 LrnΔ*χ*^2^ = 8.76 p = 0.0031* MchΔ 23/74 (31.1%)	*n* = 74 LrnΔ*χ*^2^ = 26.27 p < 0.0001* MchΔ 40/74 (54.1%)	*n* = 75 LrnΔ*χ*^2^ = 20.55 p < 0.0001* MchΔ 40/75 (53.3%)	*n* = 84 LrnΔ*χ*^2^ = 45.56 p < 0.0001* MchΔ 57/84 (67.9%)	*n* = 75 LrnΔ*χ*^2^ = 18.03 p < 0.0001* MchΔ 33/75 (44.0%)		
Lecturer cues				*n* = 83 LrnΔ*χ*^2^ = 6.76 p = 0.0093 MchΔ 34/83 (41.0%)				
Cues from student grapevine			*n* = 82 LrnΔ*χ*^2^ = 80.08 p < 0.0001* MchΔ 55/82 (67.1%)		*n* = 83 LrnΔ*χ*^2^ = 56.13 p < 0.0001* MchΔ 44/83 (53.0%)		*n* = 77 LrnΔ*χ*^2^ = 6.85 p = 0.0089 MchΔ 33/77 (42.9%)	
Lack of cues					*n* = 75 LrnΔ*χ*^2^ = 5.51 p = 0.0189 MchΔ 29/75 (38.7%)	*n* = 76 LrnΔ*χ*^2^ = 1.42 p = 0.2335 MchΔ 19/76 (25.0%)		*n* = 83 LrnΔ*χ*^2^ = 9.82 p = 0.0017* MchΔ 39/83 (47.0%)
System design								
Imminence of assessment		*n* = 83 LrnΔ*χ*^2^ = 122.11 p < 0.0001* MchΔ 74/83 (89.2%)					*n* = 83 LrnΔ*χ*^2^ = 15.99 p = 0.0001* MchΔ 37/83 (44.6%)	*n* = 83 LrnΔ*χ*^2^ = 33.09 p < 0.0001* MchΔ 54/83 (65.1%)
Pattern of scheduling						*n* = 74 LrnΔ*χ*^2^ = 0.32 p = 0.5690 MchΔ 28/74 (37.8%)		
Prevailing workload							*n* = 75 LrnΔ*χ*^2^ = 0.03 p = 0.8618 MchΔ 32/75 (42.7%)	*n* = 82 LrnΔ*χ*^2^ = 18.33 p < 0.0001* MchΔ 47/82 (57.3%)

### Role of mechanism factors

All mechanism factors were selected more or less frequently as the one option respondents felt best applied to them in a given scenario (Table 4). The proportion of respondents who changed the mechanism factor they selected varied from 25.0% to 89.2% across scenarios (Table 3). Across all item pairs, three mechanism factors (i.e. agency [f], *n* = 815, 24.5%; response efficacy [c], *n* = 792, 23.8%, and response value [e], *n* = 508, 15.3%) accounted for 63.7% of responses. These results support the role of all subcomponents of the mechanism in the model.

**Table 4:** Frequency at which mechanism factors were selected overall

Mechanism factor	Item wording	*n*	%
(a) Impact appraisal	Nothing bad will happen to me academically if I do this	281	8.5
(b) Impact severity	There is too much at stake to risk doing badly or failing	337	10.1
(c) Response efficacy	Doing this is necessary to get a result I’m comfortable with	792	23.8
(d) Response costs	It’s not worthwhile letting my life get out of balance	227	6.8
(e) Response value	It is important to me to do well: I don’t want to do badly or fail	508	15.3
(f) Agency	I believe that I’m capable of doing well enough under these circumstances if I do this	815	24.5
(g) Normative beliefs: peers	I have heard from other students that if I do this, I will do well enough	131	3.9
(h) Normative beliefs: lecturers	Lecturers emphasise the importance of this	68	2.0
(i) Motivation to comply with normative beliefs: peers	It is worth taking guidance from my fellow students	102	3.1
(j) Motivation to comply with normative beliefs: lecturers	I value the opinion of lecturers who teach this module	61	1.8
	Total	3322	100

## Discussion

The purpose of this study was to continue a process of validation of a model explaining the pre-assessment learning effects of consequential assessment. Using a broader but similar population to that in which the model was derived, we demonstrated predictable associations between assessment factors and learning effects for 13 of 21 associations that featured least commonly in the original study, even under the stringent conditions of assigning significance using a Bonferroni adjusted p-value. These results may represent the first step in falsifying the role of the eight associations for which we could not find support for model-based predictions. For now, however, we can conclude that of this subset, 13 associations do, and eight do not, hold promise to further enhance our understanding of the pre-assessment learning effects of assessment.

We also demonstrated that even in this limited subset of uncommon associations, all of the mechanism factors played a role. Furthermore, different mechanism factors were at play for different versions of a scenario for many respondents. There were instances in which, although the learning effect considered most likely to vary was selected with similar frequency between the two variants of a scenario, a majority of respondents selected different mechanism factors in the two scenarios (e.g. the association between ‘assessment criteria’ and ‘choice of resources’). This supports the model’s prediction of contextual variability for the role of mechanism factors.

This study also supports the model by way of the process of developing the instruments used. Almost all components and associations in which we were interested proved testable, insofar as we were able to devise relevant item pairs that yielded useable data. We are confident of content validity (items were developed from extensive data generated using grounded theory) and have evidence to support face validity (students pronounced scenarios and option sets to be both realistic and plausible during piloting).

Many of the subset of uncommon associations explored here are novel. Of these, two seemingly potent assessment factors for which we substantiate a role are ‘nature of assessable material’ and ‘imminence of assessment’. Their associations with some, but not all, of the limited subset of learning effects studied here have been documented before, but in a limited fashion and typically in qualitative studies.^17^,^18^,^27^–^30^ This is perhaps not surprising given that most literature relating assessment and pre-assessment learning focuses on the influence of task type (which was not included in this study of uncommon associations as we previously found associations between task type and learning effects to be common).

With regard to mechanism factors, three were prominent in this setting. The findings of other studies yield fragmentary support for the roles of appraisal of response efficacy,^17^,^19^,^28^,^30^–^35^ appraisal of response value^17^,^19^,^27^,^29^,^30^,^36^ and perceptions of agency.^17^,^19^,^30^ None of these components have been studied systematically, however. We would argue that our findings open potentially fruitful new avenues to explore.

There are methodological issues to address, however. One association in the model could not be investigated using the approach adopted for this study. Although we have been able to show a role for all mechanism factors, these data were not amenable to statistical analysis. This potentially poses a problem, given the expectation that theories should be testable,^8^ and points to the need to explore other methods to investigate the role of some components and associations in the model.

The fact that the findings reported here represent self-reported behaviour rather than observed behaviour may have resulted in the over-representation of functional learning beliefs and behaviours in comparison with dysfunctional learning beliefs and behaviours.^37^ However, responses were anonymous and the researchers were unknown to the respondents.

A further limitation is that our results potentially oversimplify the reality they represent. Respondents were limited to selecting only the one most likely learning effect and mechanism factor in each scenario. Although these results give an indication of the dominant factors at play in these scenarios for these respondents, the results of the baseline studies^9^,^16^ suggest there are likely to be several factors at play in any real-life scenario. It would be interesting, if complicated, to explore the other factors at play in any given scenario and to investigate which factors explain whether any factor is or is not at play in any given scenario.

A final limitation is that findings are limited to one medical school in one country, and that this is the same medical school in which the model was originally developed. Although we are happy that we can generalise our findings to the classes from which respondents were drawn, these findings cannot yet be generalised to other groups.

Sambell and McDowell argued that if students each construct their own version of the hidden curriculum, ‘this means that the outcomes of assessment as “lived” by students are never entirely predictable, and the quest for a “perfect” system of assessment is, in one sense, doomed from the outset’.^35^ We would rather share in Prochaska *et al.*’s^8^ optimism that the ability to situate findings in a theoretical framework will ultimately lead to more effective interventions, in this case geared towards enhancing the pre-assessment learning effects of consequential assessment.

Given the theory-building nature of this research, we are cautious about making recommendations for practice.^38^,^39^ Nonetheless, with deference to the caveats imposed by the study’s limitations, we can consider the guidance our findings give for practice. The truth of the matter is that of the subset of factors studied here, few represent factors one might want to target for interventions or, indeed, that are even amenable to interventions (e.g. ‘cues from the student grapevine’ and ‘imminence of assessment’). The value of these results lies, rather, in their furthering of our understanding of those associations between assessment factors and learning effects that have proved significant and how, even if they are not the target of an intervention, they may act as (potentially powerful) confounders to influence the results of that intervention. One point these results underline is that the relationship between assessment and pre-assessment learning effects is not limited to students’ perceptions of task demands as much literature would suggest. Furthermore, as Sambell and McDowell^35^ have intimated, students do not respond homogeneously to assessment, nor does assessment act as a homogeneous trigger for action. Our model provides a framework with which to explore this heterogeneity.

Our findings set the stage for further steps in validating the model. These will need to include efforts to explore its generalisability to other populations, such as medical students in other medical schools and countries, students studying other disciplines and postgraduate students. Future research should also explore the associations that were most common in the initial study. If those factors that do lend themselves to interventions were to become the subject of focus, experimental studies to influence learning could then be contemplated. It is hoped that as the chain of validity evidence for the model is extended with further research, practitioners and researchers will have a stronger theoretical framework with which to work when designing interventions geared towards, and research about, the pre-assessment learning effects of consequential assessment.
